# Uterine cervical adenomyoma: Case report and literature review

**DOI:** 10.1097/MD.0000000000043090

**Published:** 2025-07-04

**Authors:** Guang Shi, Yun Wang, Ying Liu, Xiu-Hua Fan, Rui-Hua Zhao

**Affiliations:** a Gynecology Department, Guang’anmen Hospital, China Academy of Chinese Medical Sciences, Beijing, China; b Pathology Department, Guang’anmen Hospital, China Academy of Chinese Medical Sciences, Beijing, China.

**Keywords:** case report, literature review, uterine cervical adenomyoma

## Abstract

**Rationale:**

Cervical adenomyoma is a rare benign tumor that is easily confused with malignant diseases of the cervix, including malignant and benign cervical diseases. Misdiagnosis results in mistakes in therapy. This study aims to enhance the understanding of cervical adenomyoma through a case report, including clinical symptoms, physical examination, and surgical photos, at the same time a systematic review of cervical adenomyoma will be done, thereby helping to avoid clinical misdiagnosis.

**Patient concerns:**

A 39-year-old woman with a cervix mass about 3 × 3 cm. The root of the tumor was located in the cervical canal, and contact bleeding was positive. The pap smear and human papillomavirus test were normal. The menstrual cycle was normal. Ultrasonography revealed that the uterus and bilateral ovaries were normal. There is no relevant family history. Cervical hyperplasia or cervical myoma was considered before surgery.

**Diagnoses:**

The patient was diagnosed with cervical adenomyoma.

**Interventions:**

Hysteroscopy was taken, and histopathology was taken after surgery.

**Outcomes:**

Histopathology revealed that the lesions were mixed cervical glands with smooth muscles.

**Lessons:**

Through the case report, we gain a better understanding of the diagnosis of cervical adenomyoma. Histopathology and immunohistochemical staining were conducive to diagnose cervical adenomyoma. A review of existing literature helps distinguish it from other benign and malignant cervical tumors, thereby reducing misdiagnosis.

## 1. Introduction

Adenomyoma of the uterus occurs when endometrial tissue is present and grows in the myometrium. The clinical symptoms include dysmenorrhea, menorrhagia, and prolonged menstruation, among others. Adenomyoma usually occurs in women of childbearing age. Cervical adenomyoma is a rare benign tumor, and histochemical staining shows that the lesion tissue of cervical adenomyoma is composed of cervical smooth muscle and endocervical epithelium. The clinical symptoms include abdominal distension, vaginal mass, abnormal vaginal discharge, and irregular vaginal bleeding, among others. Most patients have no obvious symptoms, and the mass is only occasionally found in the routine gynecological physical examination. Cervical adenomyoma is similar to polypoid hyperplasia protruding outside the cervix, to myoma and to a malignant tumor. As cervical adenomyoma is a rare disease with an unclear pathogenesis and uncertain clinical symptoms, the diagnosis requires pathology. Cervical adenomyoma is often confused with cervical adenocarcinoma, cervical hyperplasia, and cervical polyps, and it is easily misdiagnosed. If pathology indicates cervical adenomyoma, the prognosis is good after surgical resection. Here, we describe the clinical findings of a case of cervical adenomyoma and present a literature review of cervical adenomyoma.

## 2. Case report

**Figure 1.**
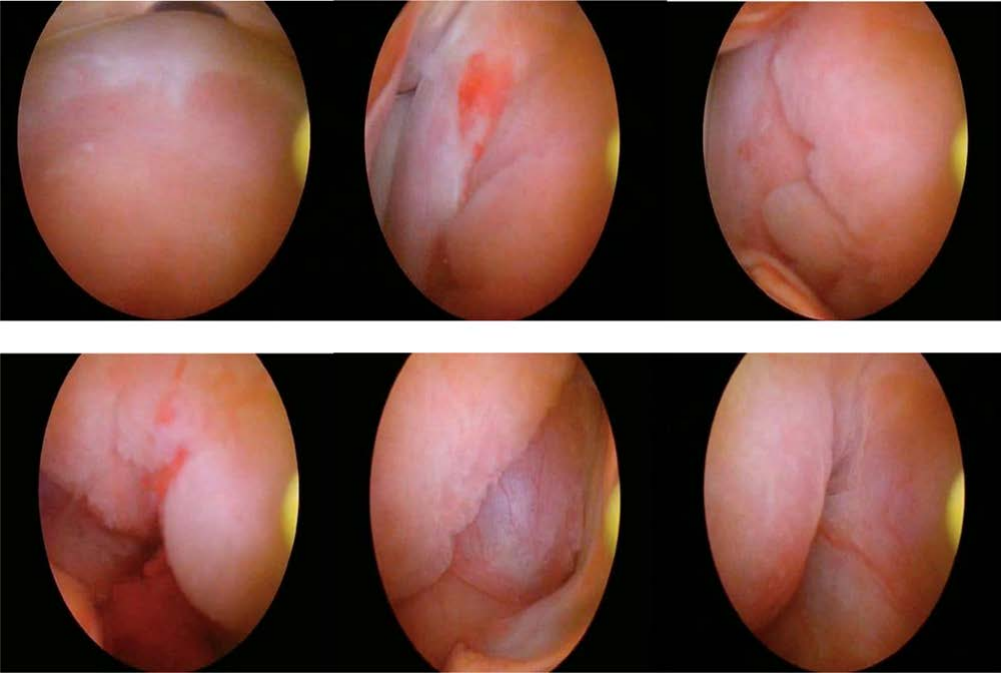
Photos of endocervical adenomyoma through hysteroscopy. Hysteroscopy showed hyperplastic tissue approximately 3 × 3 cm at the external of the cervix, mainly at the upper lip.

**Figure 2.**
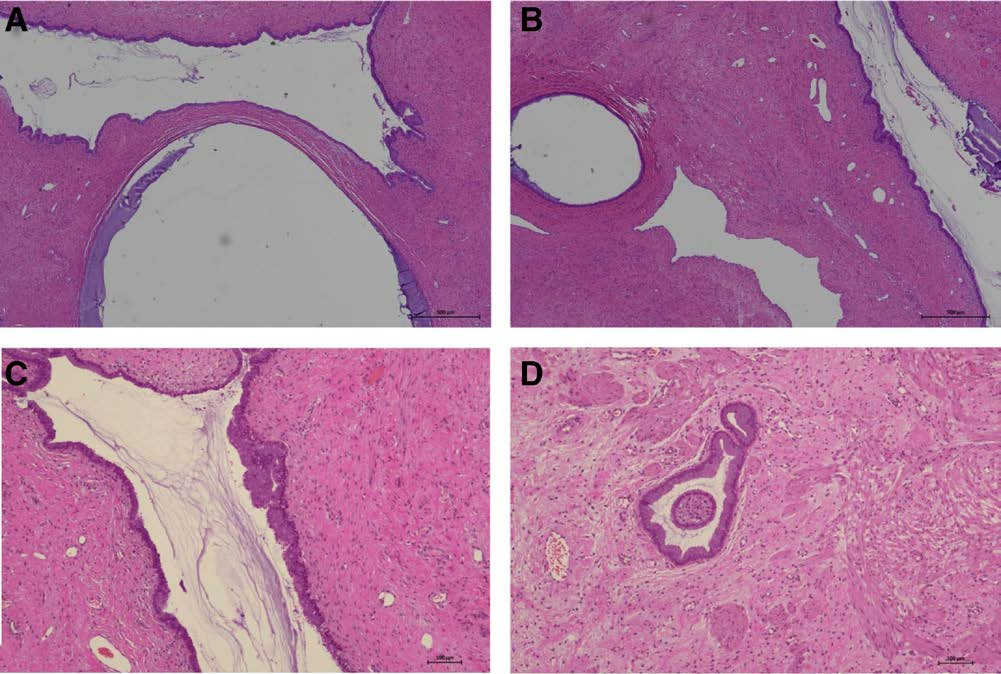
Histopathology of cervical adenomyoma sample collected by hysteroscopy. H&E staining (A) ×10, (B) ×10, (C) ×40, and (D) ×40. The tissue includes cervical glands and smooth muscle. H&E = hematoxylin and eosin.

**Figure 3.**
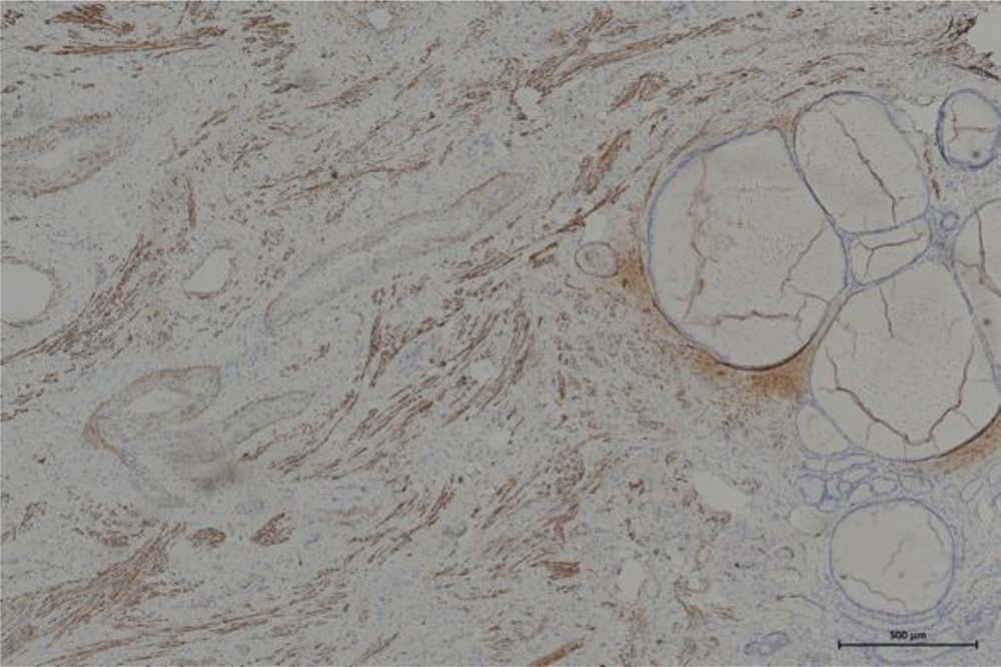
Immunohistochemical staining of desmin (×10). Immunohistochemical staining of desmin is positive in smooth muscle in cervical adenomyoma sample.

**Figure 4.**
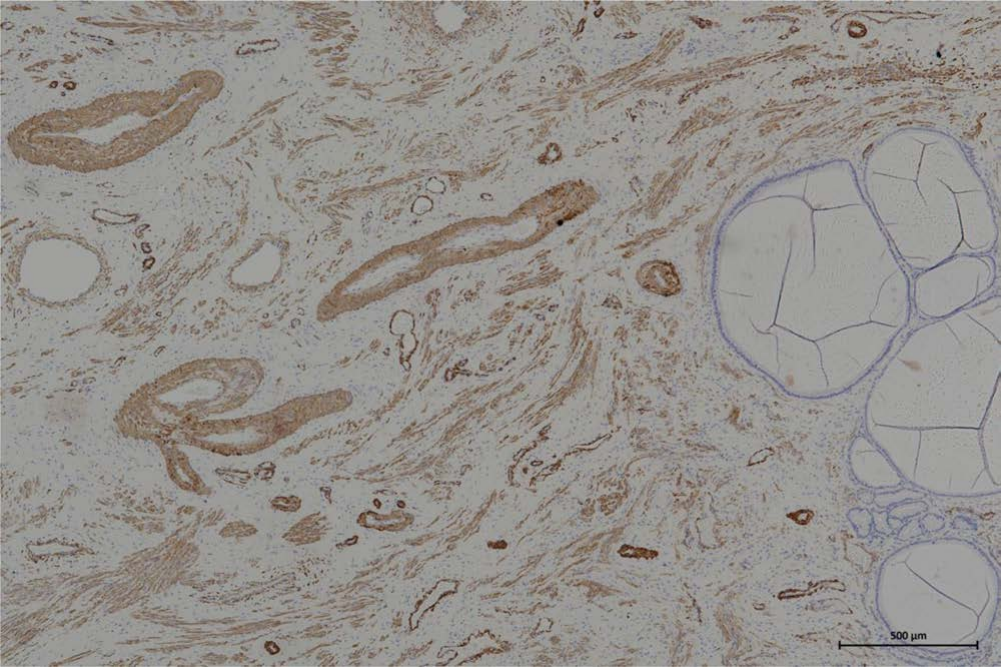
Immunohistochemical staining of smooth muscle actin (×10). Immunohistochemical staining of smooth muscle actin is positive in smooth muscle and cervical glands in cervical adenomyoma sample.

A 39-year-old Chinese woman (gravida 1, para 1) was referred to our hospital due to a cervical tumor. The cervical tumor was found to be approximately 0.5 cm before 3 years during physical examination. At that time, the patient did not do anything to address the tumor but did insist on a physical examination every year. The tumor size increased to 3 cm, similar to a polyp. The root of the tumor was located in the cervical canal, and contact bleeding was positive. The pap smear and human papillomavirus test were normal. Her menstrual cycle was normal. Ultrasonography revealed that the uterus was normal, and the thickness of the endometrium was 1.1 cm. There was no abnormal echo in the cervical canal, or in muscular layer. The bilateral ovaries were normal. There is no relevant family history. Cervical hyperplasia or cervical myoma was considered before surgery. Then, the cervical lesion was excised by hysteroscopy and loop electrosurgical excision procedure. Hysteroscopy showed hyperplastic tissue approximately 3 × 3 cm at the external of the cervix, mainly at the upper lip. Polypoid hyperplasia tissue of approximately 1.5 × 1.0 cm could be seen in the cervical canal (as shown in Fig. 1). Histopathology revealed that the lesions were mixed cervical glands with smooth muscles. Cervical adenomyoma was diagnosed (Fig. 2). Immunohistochemical staining showed negativity for carcinoembryonic antigen (CEA), and positivity for Ki-67 (+1%), desmin (Fig. 3) and smooth muscle actin (SMA) (Fig. 4). After 6 months of surgery, the patient returned to the hospital for gynecological examination, and no mass were found in cervix.

## 3. Discussion

Uterine cervical adenomyoma is a rare benign tumor.^[1,2]^ Gilks et al first reported 10 cases of benign cervical adenomyoma in 1996,^[3]^ and then some subsequent single case reports in future years. There are still a few cases of single cervical adenomyoma.^[1,2,4–6]^ In 2015, Casey et al reported a case series study of 10 cases of cervical adenomyoma.^[7]^ Irregular vaginal bleeding and vaginal prolapse are common symptoms. Some people have no specific symptoms, and only a tumor is found during physical examination. Most masses are polypoid-like. The typical histological features are a thin layer of fibrous matrix and smooth muscle around the glands. There is no interstitial connective tissue hyperplasia. Of 7 patients with cervical adenomyoma, 6 are considered to have malignant adenomas,^[7]^ which are often confused with cervical malignant adenocarcinoma, lobular endocervical glandular hyperplasia (LEGH), multiple benign cystic changes of the cervix (such as cervicitis, Nabothian cysts, endometrial hyperplasia of the cervix, and tunnel cluster).^[5]^

Cervical adenomyoma needs a definite pathological diagnosis, but it is often confused with other cervical diseases because it is a rare disease. The rarity of cervical adenomyoma makes identification very important, especially in distinguishing it from malignant adenoma. It is helpful to know the features of lesions for correct diagnosis and treatment.^[1]^ The typical histopathology of cervical adenomyoma consists of intracervical glands, smooth muscle cells and various pseudoneoplastic gland lesions.^[2]^ There is no atypia, pleomorphism or mitotic activity. The interstitial SMA staining is positive. Cervical adenomyoma is often misdiagnosed as LEGH. Patients with LEGH frequently complain of watery vaginal discharge.^[8,9]^ LEGH is almost always in the upper endocervix and is associated with enlarged cysts in the periphery.^[10,11]^ Mikami et al and Nucci et al defined the histological findings of LEGH as follows: small gland proliferation in a lobular fashion, abundant intracytoplasmic mucin of the glandular epithelium, basally located round nuclei without anaplasia and absence of destructive stromal invasion.^[12,13]^

Another category confused with cervical adenomyoma is multilocular cystic lesions, including benign and malignant lesions. Benign lesions include uterine cervicitis, endocervical hyperplasia, Nabothian cyst, and tunnel cluster. Malignant lesions include adenocarcinoma and adenoma malignum.^[14]^ Usually, benign lesions have good margination, do not invade deep into the stroma, and lack solid components.^[14]^ Uterine cervicitis is a round lesion located centrally in the cervix. Histopathology shows multiple cysts in the epithelial lining without cellular atypia. Endocervical hyperplasia is located in the endocervix and superficial layer of the cervical wall. Glands are lined by benign columnar epithelium without cellular atypia. Nabothian cysts are usually located in the uterine cervix, and there are endocervical glands. However, the occasionally extends deep into the cervical stroma. Nabothian cysts appear as a single cystic lesion or as multiple cystic lesions in the fibrous cervical stroma, are contiguous and round, and have regular boundaries. Histopathology shows mucin-filled cysts due to Nabothian cysts without cellular atypia. These dilated mucin-filled cysts show uniform architecture and are lined by flattened mucinous epithelium without atypia. Neither stratification nor mitotic figures are evident. Tunnel cluster is a type of Nabothian cyst characterized by complex multicystic dilatation of the endocervical glands.^[15]^ Tunnel cluster is a benign pseudoneoplastic glandular lesion of the cervix and is characterized as type A (noncystic) or type B (cystic).^[16]^ Histopathology shows multiple mucus-filled cysts and lobular proliferation of endocervical glands with side channels. These lesions are close to the endocervical canal and are lobular, with 1 or more discrete foci of cystically dilated endocervical glands. Neither atypia nor stromal desmoplasia is found. There is no smooth muscle proliferation in endocervical hyperplasia. Histopathology showed the presence of glands in normal or proliferative cervical stroma. Microglandular hyperplasia, tunnel cluster and cervical glandular hyperplasia do not form a gross mass, but are microscopic manifestations. However, these benign lesions may have solid features and may be similar to malignant lesions. Early identification of malignant cystic lesions such as malignant adenocarcinoma and benign cystic lesions is very important, but very difficult.

Adenocarcinoma is a subtype of cervical carcinoma that arises from the columnar epithelium of the endocervical glands. Adenocarcinoma accounts for 10% to 15% of all cervical carcinomas. Adenocarcinoma consists of mucin-producing endocervical cells and is usually located in the endocervical canal rather than the external cervix. Histopathology shows multiple cysts within the epithelial lining. Malignant epithelium lacks stroma within the single gland profile. Cellular atypia is evident. Adenoma malignum, also known as minimal-deviation adenocarcinoma, is a special subtype of mucinous adenocarcinoma of the cervix. Pathologic analysis shows conjugation of small cystic spaces lined predominantly by mucin-containing columnar epithelial cells with cystic spaces filled with mucin. Most glands have cellular atypia and structural dysplasia. Histopathology shows multilocular cystic lesions composed of a single layer of columnar cells that resemble normal endocervical glands. However, most glands have cellular atypia and structural dysplasia with multiple lobulation hairpin shapes.^[14]^ Immunohistochemical staining of periodic acid–Schiff, Alcian blue, m-ggmc-1, CEA, p53, Ki-67, HIK 1083, estrogen receptor-positive, and MUC6 may be helpful for diagnosis.^[2,5,7,17]^

This case of endocervical adenomyoma was found in the physical examination, and showed an increasing trend, without obvious clinical symptoms. Physical examination showed that the mass was large, was similar to cervical glandular hyperplasia and was a cervical myoma. The diagnosis considered before surgery may be endocervical glandular hyperplasia or cervical myoma. After surgery, histopathology showed proliferating cervical epithelial glands with smooth muscle tissue, which was considered cervical adenomyoma. Immunohistochemical staining showed CEA negativity, an increase in Ki-67 to 1%, and desmin and SMA positivity. The SMA of smooth muscle cells in normal cervical glands is typically negative, but in this case, SMA was positive. The histopathology showed a mixture of proliferating cervical glands and smooth muscle. For further identification of benign and malignant tumors, immunochemical staining of CEA and Ki-67 was performed. Cina et al described a moderate combination of cytoplasmic CEA reactivity and the labeled Ki-67 proliferation index as diagnostic evidence for cervical malignancies, including malignant adenomas.^[18]^ CEA-positive staining is considered to identify malignant tumors. However, some benign glands may also be positive. In this case, CEA reactivity was negative, and the Ki-67 index was very low. Therefore, it was diagnosed as benign cervical adenomyoma. Hysteroscopy was helpful in avoiding a misunderstanding in this case. But the limitation of this case report is the absence of preoperative pelvic magnetic resonance imaging. Magnetic resonance imaging could have yielded valuable noninvasive diagnostic information to aid in differential diagnosis. Knowing the characteristics of cervical adenomyoma is helpful for accurate diagnosis and differentiation from malignant adenoma. Therefore, the results of histopathology and immunoactivity should be combined with macroscopic and microscopic results to avoid confusing this rare tumor with malignant adenoma.^[4]^

## 4. Conclusion

This study enhances the understanding of cervical adenomyoma through a case report, including clinical symptoms, physical examination and surgical photos, at the same time a systematic review about cervical adenomyoma has be done. Key differentiation points from other cervical malignant adenocarcinoma, LEGH, and multiple benign cystic changes of the cervix, such as cervicitis, Nabothian cysts, endometrial hyperplasia of the cervix, and tunnel cluster, have been identified. It will help clinical doctors better diagnose cervical adenomyoma and reduce misdiagnoses.

## Author contributions

**Surgery procedure:** Xiu-Hua Fan, Ying Liu, Guang Shi.

**Methodology:** Yun Wang.

**Writing – original draft:** Guang Shi.

**Writing – review & editing:** Xiu-Hua Fan, Rui-Hua Zhao.
